# Serum high mobility group box-1 levels associated with cardiovascular events after lower extremity revascularization: a prospective study of a diabetic population

**DOI:** 10.1186/s12933-022-01650-1

**Published:** 2022-10-16

**Authors:** Maria Margherita Rando, Federico Biscetti, Andrea Leonardo Cecchini, Elisabetta Nardella, Maria Anna Nicolazzi, Flavia Angelini, Roberto Iezzi, Luis H. Eraso, Paul J. Dimuzio, Dario Pitocco, Antonio Gasbarrini, Massimo Massetti, Andrea Flex

**Affiliations:** 1 Cardiovascular Internal Medicine Unit, Fondazione Policlinico Universitario A. Gemelli IRCCS, Largo Agostino Gemelli 8, 00168 Rome, Italy; 2 Università Cattolica del Sacro Cuore, Largo Francesco Vito 1, 00168 Rome, Italy; 3 Radiology Unit, Fondazione Policlinico Universitario A. Gemelli, IRCCS, Largo Agostino Gemelli 8, 00168 Rome, Italy; 4 Division of Vascular and Endovascular Surgery, Thomas Jefferson University, Philadelphia, PA USA; 5 Diabetology Unit, Fondazione Policlinico Universitario A. Gemelli, IRCCS, Largo Agostino Gemelli 8, 00168 Rome, Italy; 6 Department of Medical and Surgical Sciences, Fondazione Policlinico Universitario A. Gemelli IRCCS, Largo Agostino Gemelli 8, 00168 Rome, Italy; 7 Department of Cardiovascular Sciences, Fondazione Policlinico Universitario A. Gemelli IRCCS, Largo Agostino Gemelli 8, 00168 Rome, Italy

**Keywords:** Diabetes mellitus, Peripheral artery disease (PAD), Inflammation, High mobility group box-1 (HMGB-1), Lower-extremity endovascular revascularization (LER)

## Abstract

**Background:**

Peripheral arterial disease (PAD) is one of the most disabling cardiovascular complications of type 2 diabetes mellitus and is indeed associated with a high risk of cardiovascular and limb adverse events. High mobility group box-1 (HMGB-1) is a nuclear protein involved in the inflammatory response that acts as a pro-inflammatory cytokine when released into the extracellular space. HMBG-1 is associated with PAD in diabetic patients.

The aim of this study was to evaluate the association between serum HMGB-1 levels and major adverse cardiovascular events (MACE) and major adverse limb events (MALE) after lower-extremity endovascular revascularization (LER) in a group of diabetic patients with chronic limb-threatening ischemia (CLTI).

**Methods:**

We conducted a prospective observational study of 201 diabetic patients with PAD and CLTI requiring LER. Baseline serum HMGB-1 levels were determined before endovascular procedure. Data on cardiovascular and limb outcomes were collected in a 12-month follow-up.

**Results:**

During the follow-up period, 81 cases of MACE and 93 cases of MALE occurred. Patients who subsequently developed MACE and MALE had higher serum HMGB-1 levels. Specifically, 7.5 ng/mL vs 4.9 ng/mL (p < 0.01) for MACE and 7.2 ng/mL vs 4.8 ng/mL (p < 0.01) for MALE. After adjusting for traditional cardiovascular risk factors, the association between serum HMGB-1 levels and cardiovascular outcomes remained significant in multivariable analysis. In our receiver operating characteristic (ROC) curve analysis, serum HMGB-1 levels were a good predictor of MACE incidence (area under the curve [AUC] = 0.78) and MALE incidence (AUC = 0.75).

**Conclusions:**

This study demonstrates that serum HMGB-1 levels are associated with the incidence of MACE and MALE after LER in diabetic populations with PAD and CLTI.

## Background

Diabetes mellitus (DM) represents a public health problem and economic burden, with an annual cost of 327 billion dollars in the United States [1]. Peripheral arterial disease (PAD) affects approximately 20–26% of diabetic patients [2] and among cardiovascular complications of diabetes it is one of the most disabling, with a significant impact on healthcare [3]. PAD is indeed associated with a high risk of major cardiovascular events (MACE), particularly myocardial infarction, stroke and cardiovascular death, and major adverse limb events (MALE), including acute limb ischemia, major vascular amputations, and limb-threatening ischemia needing urgent revascularization [4]. In addition, patients with PAD and diabetes have a higher risk of both MACE and MALE, compared to those without diabetes, notwithstanding current treatment with antiplatelets, statins and antihypertensive drugs [2].

Treatment of PAD is based on a multidisciplinary approach, including best medical therapy, aimed at managing modifiable cardiovascular risk factors, and lower extremity revascularization in the setting of chronic limb-threatening ischemia (CLTI) [5, 6]. Among revascularization strategies, endovascular intervention has increased significantly in recent years, representing an effective treatment option for improving symptoms and preventing limb loss [5, 7, 8]. After LER, however, cardiovascular and limb events occur in a considerable percentage of patients [9–11]. Therefore, identifying biomarkers that can predict the risk of cardiovascular complications is necessary to develop a personalized follow-up approach.

In diabetic patients, outcomes after revascularization may be related to multiple mechanisms, not all of which are well defined. Diabetes accelerates the progression of atherosclerosis in several ways, including insulin resistance and hyperinsulinemia, hyperglycemia, diabetic dyslipidemia, advanced glycation end-product, reactive oxygen species, and inflammation [2]. Particularly, inflammation plays a crucial role in the development of PAD [12, 13]. In addition, C-reactive protein (CRP) is associated with poor outcomes after LER, including restenosis after angioplasty, risk of limb loss, cardiovascular events, and mortality [14–16]. Recently, we found that baseline levels of osteoprotegerin (OPG), tumor necrosis factor (TNF)-α, interleukin (IL)-6, and CRP levels were associated with adverse cardiovascular outcomes in diabetic patients with PAD and CLTI after LER [17].

Among inflammatory biomarkers associated with the atherosclerotic process and diabetic complications, high mobility group box-1 (HMGB-1) plays a crucial role in the inflammatory response responsible for vascular injury [18, 19]. HMGB-1 is a nuclear protein involved in inflammation, angiogenesis and tissue regeneration [20–24] and it has been shown that it is overexpressed in diabetic patients compared to patients without diabetes [25, 26]. HMGB-1 plays a role in DNA transcription, replication and repair in the nucleus and it acts as a signal regulator in the cytoplasm. When released in the extracellular space, it acts as a pro-inflammatory cytokine [24, 27]. HMGB-1 can be passively released after cellular injury or cellular death, and released from immune cells (monocytes, macrophages, dendritic cells, natural killer (NK) cells and endothelial cells) upon activation by lipopolysaccharide (LPS), TNF-α, IL-6, IL-1β, interferon (IFN)-γ or after tissue damage [18]. Once in extracellular space, HMGB-1 binds its receptors, receptor for advanced glycation end products (RAGE) and toll-like receptor (TLR)s, and stimulates both the inflammatory and immune response, involved in endothelial dysfunction and atherosclerotic process [18].

Different evidence shows a role of HMGB-1 in cardiovascular diseases development. It has been associated with coronary artery disease (CAD) [28–31] and it represents an independent risk factor for carotid plaque vulnerability [32]. In addition, we previously observed that levels of HMGB-1 were higher in patients with PAD and diabetes mellitus compared to those without diabetes mellitus [33]. Given available evidence, a role of HMBG-1 has been postulated in predicting cardiovascular outcomes after LER.

The aim of this study was to evaluate the association between serum levels of HMGB-1 at baseline and MACE and MALE, in diabetic patients with PAD and CLTI requiring for LER.

## Research design and methods

### Study design

The prospective cohort study aimed at investigating the association between HMGB-1 serum levels and the incidence of MACE and MALE in a population of T2DM patients with PAD and CLTI, requiring LER. The study has been approved by the Ethics Committee of the Fondazione Policlinico Universitario A. Gemelli IRCCS and it adhered to the principles of the Declaration of Helsinki. All the subjects enrolled have given informed consent to participate.

### Study population and clinical assessment

We enrolled 212 consecutive patients with T2DM and PAD needing LER and admitted to Internal Medicine Cardiovascular Unit of the Fondazione Policlinico Universitario A. Gemelli IRCCS, Roma, Italy from October 23, 2019 to June 30, 2021.

All patients over 18 years of age with a diagnosis of T2DM present for a least one year, an Ankle/Brachial Index (ABI) of less than 0.80, lower limb stenosis greater than 50% documented by Ultrasound Color Doppler (US), category 4 or 5 of PAD in accordance with the Rutherford classification [34], presence of CLTI as previously defined [17, 35, 36], and the need for endovascular treatment, were included in the study.

The exclusion criteria were pregnancy, acute infections at present or in the previous month, diabetic foot ulcers with sign of infection or osteomyelitis, previous lower-limb endovascular or surgical revascularization within the past 3 months, diabetic peripheral neuropathy ruled out as previously described [17], liver disease with a functional status B or C in accord to Child–Pugh classification, congenital or acquired thrombophilia, active autoimmune disease, active cancer, organ transplantation, life expectancy < 12 months, contraindication to antiplatelet therapy, contraindication to endovascular revascularization. In the presence of diabetic foot ulcer, the Wound, Ischemia, and foot Infection (WIfI) classification system was used to classify patients, as previously described [37, 38]. Radiological imaging was performed to exclude osteomyelitis, if suspected. Definition of PAD was in accordance with the Society for Vascular Surgery and the International Society for Cardiovascular Surgery criteria [39]. A lower-limb US evaluation was performed in all the patients involved in the study. Moreover, people with an ABI of 1.40 or higher, US was used to confirm the presence of severe stenosis of the lower limb.

Demographic, clinical and laboratory tests data were collected for all the subjects involved in the study. In particular, data about age, sex, body mass index (BMI), history of CAD, cerebrovascular disease (CVD), smoking habits, diabetes duration, antidiabetic medications, hypertension, hypercholesterolemia, renal failure (defined as an estimated glomerular filtration rate (eGRF) < 60 mL/min) were collected.

Before LER, patients were all in single antiplatelet therapy, beginning double antiplatelet therapy (DAPT) after revascularization procedure for 1 month. In particular, 189 patients were on aspirin and 12 patients were on clopidogrel at baseline. After the procedure, all patients of our cohort were on aspirin and clopidogrel. Furthermore, after LER, an aggressive lipid-lowering therapy was recommended to achieve an LDL-C target lower than 55 mg/dL, according to ESC/EAS Guidelines for the management of dyslipidemias [40]. At baseline, 129 (62.3%) patients were taking lipid-lowering drugs, among them 115 were on statin therapy, 10 on ezetimibe and 4 on PCSK9 inhibitor (PCSK9i). After the enrollment, cholesterol-lowering therapy was modified in all patients who did not meet treatment goal by changing the type or the dose of statin or adding another drug (e.g., ezetimibe, PCSK9i).

### Lower-limb endovascular revascularization and follow-up after the procedure

Lower-limb revascularization through balloon angioplasty and/or stenting was performed as previously described [17, 35, 36]. Revascularization was considered successful if the residual stenosis of the treated arterial vessel was less than 30%.

In accordance with the definitions of the Society of Interventional Radiology, no complications related to the endovascular procedure were recorded [41].

The incidence of MACE and MALE were assessed in a 12-month follow-up period, with evaluation of the patients at 1, 3, 6 and 12 months after LER procedure. MACE was defined as composite of cardiovascular death, stroke, or myocardial infarction [42]. MALE was defined as a composite of acute limb ischemia, major vascular amputations, and limb-threatening ischemia leading to urgent revascularization [17, 42].

### Blood test and biochemical assays

Blood tests were collected after an overnight fast at baseline, before LER, in all the patients enrolled in the study. Total cholesterol, LDL cholesterol, triglycerides, glucose, creatinine, and glycated hemoglobin were assessed. EGFR was calculated by modification of diet in renal disease (MDRD) formula. Serum was separated by centrifugation of blood samples and was stored at − 80 °C before every analysis. Serum HMGB-1 levels were determined by a commercially available ELISA kit (HMGB-1 ELISA kit II; Shino-Test Corporation, Tokyo) according to its protocol. The detection limit for HMGB-1 was 0.2 ng/mL with an inter-assay coefficient of variation (CV) < 10% [33]. The serum levels were measured twice for each patient and the results were averaged.

### Statistical analysis

A descriptive analysis of demographic and clinical data was summarized as means (standard deviations) for continuous variables and counts (percentages) for categorical variables.

The baseline characteristics of the population were compared using the chi-square and t-tests, when appropriate. A log transformation was applied to the not normally distributed variables prior to performing further analysis. Serum HMGB-1 levels were compared with Mann–Whitney, Kruskal–Wallis and Dunn’s Multiple Comparison, when appropriate. A multivariable stepwise logistic regression analysis was performed, adjusted for traditional cardiovascular risk factors and HMGB-1 levels. The area under the receiver operating characteristics (ROC) curve was calculated to test the predictive discrimination of serum HMGB-1 levels for MACE and MALE. A second model to test the predictive discrimination HMGB-1 levels and traditional cardiovascular risk factors (sex, age, diabetes duration, BMI, smoking status, hypertension, previous CAD and CVD history, treatment, total cholesterol, LDL cholesterol, triglycerides, fasting blood glucose (FBG), HbA1c) for MACE was made, comparing the areas under the ROC curves through the roccomp function in STATA software.

MACE-free survival, according to HMGB-1 levels, was evaluated using Kaplan-Mayer method and compared using the Log-Rank test. All analyses were performed using STATA version 14.0 for MacOS (Statistics/Data Analysis, Stata Corporation, College Station, TX, USA) and SPSS version 25.0 for MacOS (IBM Corporation, Armonk, NY, USA). Statistical significance was established at p < 0.05.

## Results

### Characteristics of the study population

Of the 212 patients who underwent endovascular intervention, 11 (5.2%) had a poor primary outcome and were excluded from the study follow-up.

Overall, 201 diabetic patients with PAD and CLTI were enrolled in the study. The mean age (SD) of the subjects involved was 74.9 (± 8.9) years and 150 (74.6%) of them were men. The average T2DM duration was 11.4 (± 0.8) years. Current smokers were 103 (51.2%), former smokers were 68 (33.8%) while people who had never smoked were 31 (15.4%). Regarding PAD disease severity categories, Rutherford 4 was found in 54.2% (109 patients) of the population and Rutherford 5 was present in 45.8% (92 patients) one, while WIfI010 was detected in 30.3% (61 patients), WIfI020 in 25.4% (51 patients), WIfI110 in 24.4% (49 patients) and WIfI120 in 19.9% (40 patients) of the population. It should be noted that three patients with proximal lesions were considered as Rutherford 5, according to the definition of Rutherford classification [34], and as WIfI0, according to the definition of WIfI classification [37].

Mean LDL-C levels were 112.2 mg/dL (± 15.4) and mean glycated hemoglobin levels were 8.9% (± 0.7). Mean HMGB-1 serum levels were 5.9 ng/mL (± 2.7).

The complete demographic and clinical characteristics of the population are described in Table 1.

**Table 1:** Demographic characteristics and clinical data of the study cohort at baseline

Number of patients	201
Men/female, n	150:51
Age, years ± SD	74.9 ± 8.9
Diabetes duration, years ± SD	11.4 ± 0.8
BMI, Kg/m^2^ ± SD	26.7 ± 0.6
Smoking (current), n (%)	103 (51.2)
Smoking (former), n (%)	68 (33.8)
Never smoked, n (%)	31 (15.4)
Hypertension, n (%)	146 (72.6)
Hypercholesterolemia, n (%)	109 (54.2)
CAD, n (%)	93 (46.3)
CVD, n (%)	99 (49.2)
Oral antidiabetic agents, n (%)	89 (44.3)
Insulin, n (%)	147 (73.1)
ABI, ± SD	0.6 ± 0.1
Rutherford II-4, n (%)	109 (54.2)
Rutherford III-5, n (%)	92 (45.8)
WIfI010, n (%)	61 (30.3)
WIfI020, n (%)	51 (25.4)
WIfI110, n (%)	49 (24.4)
WIfI120, n (%)	40 (19.9)
HbA1c, % ± SD	8.9 ± 0.7
FBG, mg/dL ± SD	127.9 ± 9.7
Total cholesterol, mg/dL ± SD	218.9 ± 22.3
LDL cholesterol, mg/dL ± SD	112.4 ± 15.4
Triglycerides, mg/dL ± SD	172.5 ± 8.5
eGFR, mL/min/1.73m^2^ ± SD	72.6 ± 10.5
HMGB-1, ng/mL ± SD	5.9 ± 2.7

Data are reported as means (standard deviation) for continuous variables and numbers (percentages) for categorical variables

BMI, body mass index; CAD, coronary artery disease; CVD, cerebrovascular disease; ABI, ankle brachial index; WIfI, wound, ischemia, foot infection; FBG, fasting blood glucose; eGFR, estimated glomerular filtration rate; HMGB-1, high mobility group box 1

### HMGB-1 serum levels and incidence of MACE during the follow-up period after LER

During the follow-up period of 12 months, 81 patients had a MACE after the revascularization procedure. In particular, we observed 42 myocardial infarctions, 33 strokes and 14 deaths.

There were no differences between patients with MACE and without MACE in terms of age (*p* = 0.44), sex (*p* = 0.13), BMI (*p* = 0.22), diabetes duration (*p* = 0.92), history of high blood pressure (*p* = 0.48) and hypercholesterolemia (*p* = 0.40), history of CAD (*p* = 0.66) and CVD (*p* = 0.75), antidiabetic oral medications (*p* = 0.74) and insulin (*p* = 0.57), total cholesterol levels (*p* = 0.67), LDL-C levels (*p* = 0.836), ABI (*p* = 0.75), Rutherford category (II-4 *p* = 0.40, III-5 *p* = 0.40), glycated hemoglobin levels (*p* = 0.30) and renal function (*p* = 0.67).

**Fig. 1:**
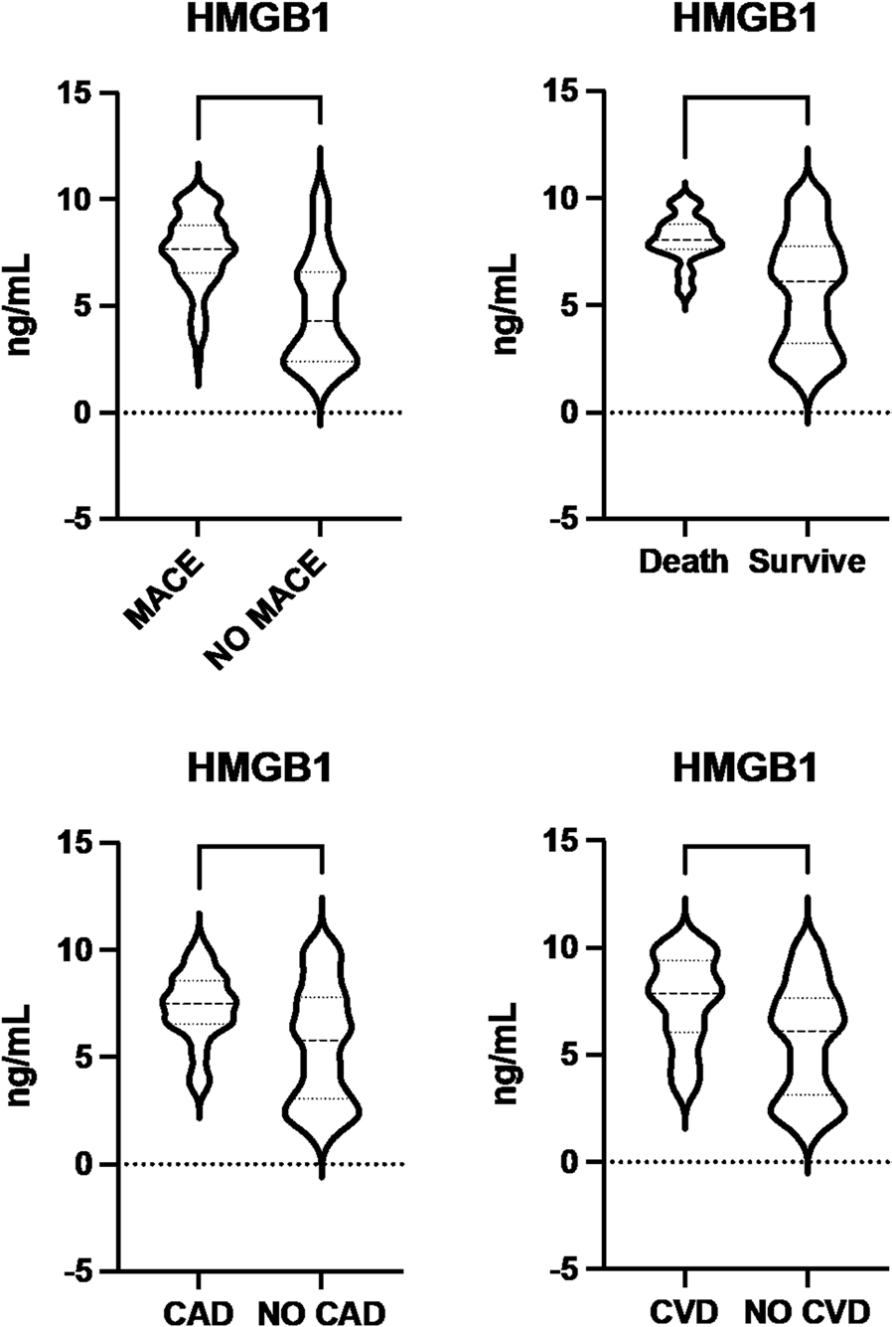
HMGB-1 levels according to MACE, mortality, coronary artery disease (CAD), cerebrovascular artery disease (CVD). On the violin plots, central line represents the median, upper line represents the upper interquartile range (IQR) and the lower line represents the lower IQR. ****p < 0.0001, **p < 0.01

Patients with MACE had higher HMGB-1 serum levels (7.5 ± 1.9 ng/mL vs 4.9 ± 2.6 ng/mL, *p* < 0.01) (Fig. 1), were active smokers (56, *p* < 0.01) and had higher levels of triglycerides (174.5 ± 8. 3 mg/dL, *p* < 0.01), compared to patients without MACE. Patients without MACE were former smokers (50, *p* < 0.01) and never smokers (24, *p* = 0.03) compared to patients with MACE. The complete characteristics of the population with and without MACE are described in Table 2.

**Table 2:** Demographic and clinical data of study participants without or with MACE

	NO MACE (n = 120)	MACE (n = 81)	p value
Men/female, n	85:35	65:16	0.13
Age, years ± SD	74.5 ± 9.3	75.5 ± 8.3	0.44
Diabetes duration, years ± SD	11.4 ± 0.9	11.4 ± 0.8	0.92
BMI, Kg/m^2^ ± SD	26.7 ± 0.6	26.8 ± 0.7	0.22
Smoking (current), n (%)	47 (39.2)	56 (69.1)	< 0.01
Smoking (former), n (%)	50 (41.7)	18 (22.2)	< 0.01
Never smoked, n (%)	24 (20.0)	7 (8.6)	0.03
Hypertension, n (%)	85 (70.8)	61 (75.3)	0.48
Hypercholesterolemia, n (%)	68 (56.7)	41 (50.6)	0.40
CAD, n (%)	54 (45.0)	39 (48.1)	0.66
CVD, n (%)	58 (48.3)	41 (50.6)	0.75
Oral antidiabetic agents, n (%)	52 (43.3)	37 (45.7)	0.74
Insulin, n (%)	86 (71.7)	61 (75.3)	0.57
ABI, ± SD	0.6 ± 0.1	0.6 ± 0.1	0.75
Rutherford II-4, n (%)	68 (56.7)	41 (50.6)	0.40
Rutherford III-5, n (%)	52 (43.3)	40 (49.4)	0.40
WIfI010, n (%)	32 (26.7)	29 (35.8)	0.17
WIfI020, n (%)	35 (29.2)	16 (19.8)	0.13
WIfI110, n (%)	26 (21.7)	23 (28.4)	0.27
WIfI120, n (%)	27 (22.5)	13 (16.0)	0.26
HbA1c, % ± SD	8.9 ± 0.7	8.8 ± 0.7	0.30
FBG, mg/dL ± SD	128.0 ± 10.0	127.8 ± 9.1	0.89
Total cholesterol, mg/dL ± SD	219.4 ± 21.5	218.1 ± 23.6	0.67
LDL cholesterol, mg/dL ± SD	112.0 ± 15.4	113.0 ± 15.6	0.70
Triglycerides, mg/dL ± SD	171.1 ± 8.3	174.5 ± 8.3	< 0.01
eGFR, mL/min/1.73m^2^ ± SD	72.3 ± 10.5	73.0 ± 10.6	0.67
HMGB-1, ng/mL ± SD	4.9 ± 2.6	7.5 ± 1.9	< 0.01

Statistical test performed with Student’s t-test or with Chi square test, when appropriate

BMI, body mass index; CAD, coronary artery disease; CVD, cerebrovascular disease; ABI, ankle brachial index; WIfI, wound, ischemia, foot infection; FBG, fasting blood glucose; eGFR, estimated glomerular filtration rate; HMGB-1, high mobility group box 1

HMGB-1 levels were analyzed in association with every MACE composite outcome: cardiovascular death, myocardial infarction and stroke. In particular, patients who died for a cardiovascular reason had higher levels of HMGB-1 compared to patients who survived (8.098 ± 1.103 ng/mL vs 5.758 ± 2.647 ng/mL, *p* = 0.0012). Patients with myocardial infarction had HMGB-1 levels of 7.313 ± 1.650 ng/mL and those without myocardial infarction 5.553 ng/mL ± 2.728 (*p* = 0.0001). Patients with stroke had HMGB-1 levels of 7.537 ± 2.074 ng/mL than those without stroke 5.604 ng/mL ± 2.624 (*p* < 0.0001) (Fig. 1).

**Fig. 2:**
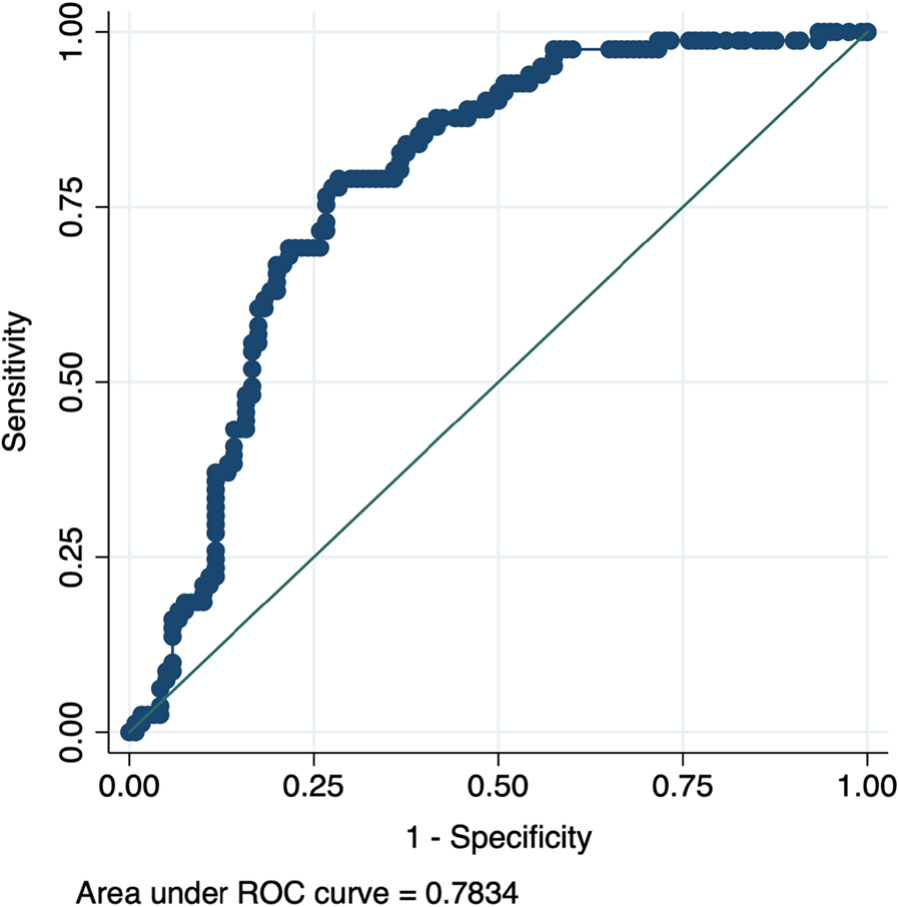
Receiver operating characteristic (ROC) curve analysis to predict incidence of MACE related to HMGB1 levels showing area under the curve (AUC). p < 0.01

To predict the incidence of MACE based on serum HMGB-1 levels, a ROC curve was elaborated. The area under the curve (AUC) was 0.7834. (95% CI 0.7195, 0.8473) (Fig. 2).

**Fig. 3:**
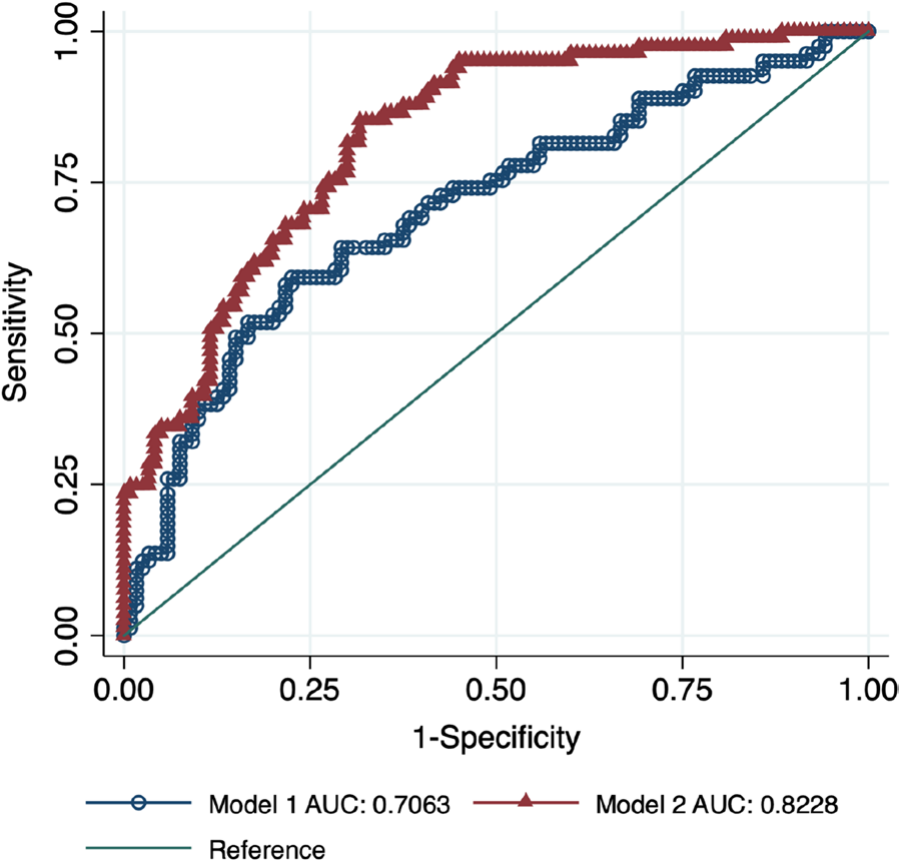
Receiver operating characteristic (ROC) curves comparing the performance of a model without (Model 1) and with (Model 2) HMGB-1 in predicting MACE. The true-positive rate (sensitivity) is plotted as a function of the false-positive rate (1—Specificity). p < 0.01

A second model to predict the incidence of MACE based on serum HMGB-1 levels and traditional cardiovascular risk factors (sex, age, diabetes duration, BMI, smoking status, hypertension, previous CAD and CVD history, treatment, total cholesterol, LDL cholesterol, triglycerides, fasting blood glucose (FBG), HbA1c) was made. The comparison of ROC curves between the baseline model with only serum HMGB-1 levels and the second model including serum HMGB-1 levels and traditional cardiovascular risk factors showed that including traditional cardiovascular risk factors improved the prediction of MACE, with an area under the ROC curve of 0.7063 (95% CI 0.6315, 0.7810) for the baseline model and 0.822 (95% CI 0.7657, 0.8799) for the second model (Fig. 3).

Based on serum HMGB-1 levels, a MACE free survival analysis was performed using a Kaplan-Mayer curve.

**Fig. 4:**
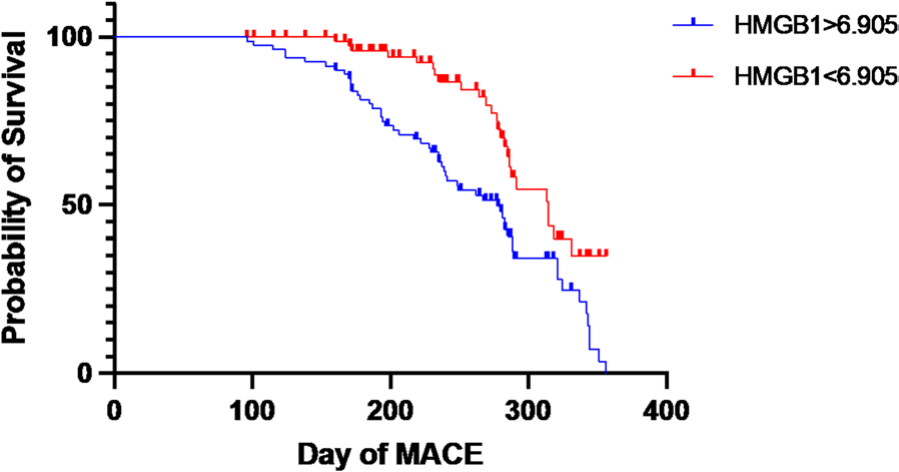
MACE free survival according to HMGB-1 levels was evaluated using Kaplan-Mayer method and compared using the Log-Rank test. Serum HMGB-1 levels higher than 6.905 ng/mL was associated with of a higher incidence of MACE compared to serum HMGB-1 levels less than 6.905 ng/mL with a sensitivity of 69.14% and a specificity of 78.33% (*p* = 0.0005)

Subjects with serum HMGB-1 levels higher than 6.905 ng/mL had a higher incidence of MACE compared to subjects with HMGB-1 serum levels less than 6.905 ng/mL with a sensitivity of 69.14% and a specificity of 78.33% (*p* = 0.0005) (Fig. 4).

After adjustment for traditional cardiovascular risk factors, a multivariable analysis was elaborated, showing that ABI (*p* = 0.001, 95% CI 0.417, 1.549) and HMGB-1 serum levels (*p* < 0.000, 95% CI 0.082, 0.131) were independent risk factors for MACE in patients with PAD who underwent LER (Table 3).

**Table 3:** Multivariable logistic regression for MACE

	Coef.	St.Err.	t-value	p-value	[95% Conf Interval]		Sig.
Men/female	0.089	0.071	1.25	0.213	− 0.051	0.230	
Age, years	0.003	0.003	0.90	0.370	− 0.004	0.009	
Diabetes duration	− 0.020	0.034	− 0.59	0.559	− 0.088	0.048	
BMI	0.066	0.047	1.41	0.161	− 0.026	0.158	
Smoking (current)	− 0.180	0.437	− 0.41	0.681	− 1.042	0.683	
Smoking (former)	− 0.357	0.434	− 0.82	0.412	− 1.213	0.499	
Never smoked	− 0.422	0.429	− 0.98	0.326	− 1.269	0.424	
Hypertension	− 0.001	0.066	− 0.01	0.988	− 0.131	0.129	
Hypercholesterolemia	− 0.095	0.060	− 1.58	0.116	− 0.213	0.024	
CAD	0.078	0.060	1.31	0.190	− 0.039	0.196	
CVD	− 0.059	0.062	− 0.95	0.341	− 0.182	0.063	
Oral antidiabetic ag	− 0.015	0.061	− 0.24	0.812	− 0.136	0.107	
Insulin	0.012	0.068	0.17	0.864	− 0.123	0.147	
ABI	0.983	0.287	3.43	0.001	0.417	1.549	**
Rutherford II-4	− 0.017	0.071	− 0.24	0.811	− 0.158	0.124	
Rutherford III-5	0.000						
WIfI010	0.180	0.092	1.95	0.053	− 0.002	0.363	
WIfI020	0.069	0.094	0.73	0.466	− 0.117	0.256	
WIfI110	0.136	0.096	1.42	0.158	− 0.053	0.325	
WIfI120	0.000						
HbA1c	− 0.062	0.045	− 1.37	0.171	− 0.151	0.027	
FBG	0.001	0.003	0.34	0.734	− 0.005	0.007	
Total cholesterol	− 0.002	0.001	− 1.44	0.152	− 0.005	0.001	
LDL cholesterol	0.002	0.002	0.93	0.356	− 0.003	0.007	
Triglycerides	0.006	0.004	1.71	0.088	− 0.001	0.013	
eGFR	0.002	0.003	0.77	0.444	− 0.003	0.008	
HMGB-1	0.107	0.012	8.56	0.000	0.082	0.131	**
Constant	− 3.008	1.779	− 1.69	0.093	− 6.520	0.503	
Mean dependent var	0.403	SD dependent var	0.492				
R-squared	0.424	Number of obs	201.000				
F-test	5.150	Prob > F	0.000				
Akaike crit. (AIC)	225.220	Bayesian crit. (BIC)	311.105				

**p < 0.01

### HMGB-1 serum levels and incidence of MALE during the follow-up period after LER

During the follow-up period of 12 months, 93 patients had a MALE after the revascularization procedure.

There were no differences between patients with MALE and without MALE in terms of age (*p* = 0.19), sex (*p* = 0.40), BMI (*p* = 0.63), diabetes duration (*p* = 0.77), history of high blood pressure (*p* = 0.62), hypercholesterolemia (*p* = 0.31), history of CAD (*p* = 0.56), history of CVD (*p* = 0.53), antidiabetic oral medications (*p* = 0.95) and insulin (*p* = 0.06), LDL-C levels (*p* = 0.61), ABI (*p* = 0.88), Rutherford category (4 *p* = 0.68, 5 *p* = 0.68), glycated hemoglobin levels (*p* = 0.10) and renal function (*p* = 0.95).

**Fig. 5:**
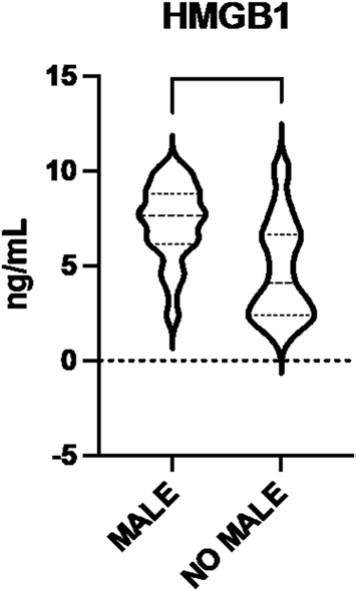
HMGB-1 levels according to MALE. On the violin plots, central line represents the median, upper line represents the upper interquartile range (IQR) and the lower line represents the lower IQR. ****p < 0.0001

Patients with MALE had higher serum HMGB-1 levels (7.2 ± 2.0 ng/mL vs 4.8 ± 2.6 ng/mL, *p* < 0.01) (Fig. 5), were active smokers (56, *p* < 0.02) and they had higher levels of total cholesterol (223.0 ± 20.7 mg/dL, *p* = 0.01), triglycerides (173.76 ± 8.5 mg/dL, *p* = 0.04), compared to patients without MALE.

The complete characteristics of the population with and without MALE are described in Table 4.

**Table 4:** Demographic and clinical data of study participants without or with MALE

	NO MALE (n = 108)	MALE (n = 93)	p value
Men/female, n	78:30	72:21	0.40
Age, years ± SD	74.1 ± 9.3	75.8 ± 8.4	0.19
Diabetes duration, years ± SD	11.4 ± 0.8	11.4 ± 0.8	0.77
BMI, Kg/m^2^ ± SD	26.7 ± 0.6	26.7 ± 0.7	0.63
Smoking (current), n (%)	47 (43.5)	56 (60.2)	0.02
Smoking (former), n (%)	46 (42.6)	22 (23.7)	< 0.01
Never smoked, n (%)	16 (14.8)	15 (16.1)	0.80
Hypertension, n (%)	80 (74.1)	66 (71.0)	0.62
Hypercholesterolemia, n (%)	55 (50.9)	54 (58.1)	0.31
CAD, n (%)	51 (47.2)	41 (44.1)	0.56
CVD, n (%)	51 (47.2)	48 (51.6)	0.53
Oral antidiabetic agents, n (%)	48 (44.4)	41 (44.1)	0.95
Insulin, n (%)	73 (67.6)	74 (79.6)	0.06
ABI, ± SD	0.6 ± 0.1	0.6 ± 0.1	0.88
Rutherford II-4, n (%)	60 (55.6)	49 (52.7)	0.68
Rutherford III-5, n (%)	48 (44.4)	44 (47.3)	0.68
WIfI010, n (%)	36 (33.3)	25 (26.9)	0.32
WIfI020, n (%)	26 (24.1)	25 (26.9)	0.64
WIfI110, n (%)	26 (24.1)	23 (24.7)	0.91
WIfI120, n (%)	20 (18.5)	20 (21.5)	0.60
HbA1c, % ± SD	8.8 ± 0.7	8.9 ± 0.7	0.10
FBG, mg/dL ± SD	128.6 ± 9.8	127.1 ± 9.3	0.27
Total cholesterol, mg/dL ± SD	215.3 ± 23.1	223.0 ± 20.7	0.01
LDL cholesterol, mg/dL ± SD	111.9 ± 15.5	113.0 ± 15.4	0.61
Triglycerides, mg/dL ± SD	171.4 ± 8.3	173.8 ± 8.5	0.04
eGFR, mL/min/1.73m^2^ ± SD	72.5 ± 10.7	72.6 ± 10.4	0.95
HMGB-1, ng/mL ± SD	4.8 ± 2.6	7.2 ± 2.0	< 0.01

Statistical test performed with Student’s t-test or with Chi square test, when appropriate

BMI, body mass index; CAD, coronary artery disease; CVD, cerebrovascular disease; ABI, ankle brachial index; WIfI, wound, ischemia, foot infection; FBG, fasting blood glucose; eGFR, estimated glomerular filtration rate; HMGB-1, high mobility group box 1

**Fig. 6:**
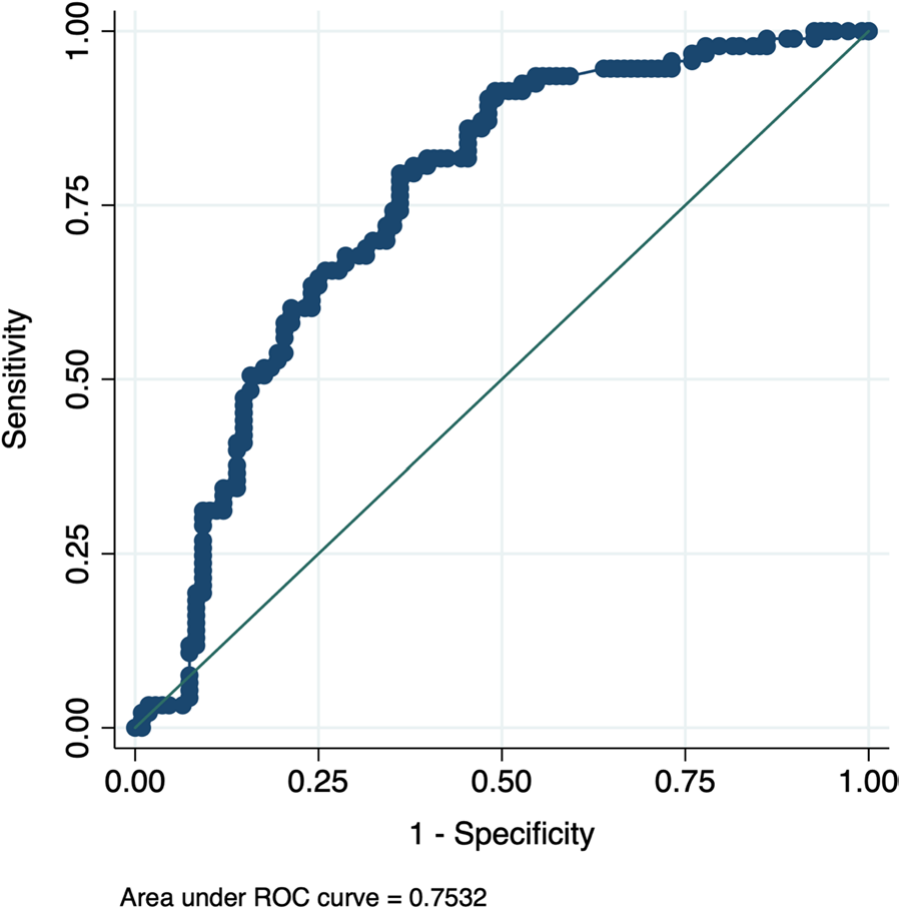
Receiver operating characteristic (ROC) curve analysis to predict incidence of MALE related to HMGB-1 showing area under the curve (AUC). p < 0.01

To predict the incidence of MALE based on HMGB-1 serum levels, a ROC curve was elaborated. The area under the curve (AUC) was 0.7532 (95% CI 0.6852, 0.8211) (Fig. 6).

After adjustment for traditional cardiovascular risk factors, a multivariable analysis was elaborated, showing that use of insulin (*p* = 0.042, 95% CI 0.006, 0.297), ABI (*p* = 0.015, 95% CI 0.153, 1.373), glycated hemoglobin (*p* = 0.002, 95% CI 0.054, 0.245), total cholesterol (*p* = 0.017, 95% CI 0.001, 0.007) and serum HMGB-1 levels (*p* < 0.000, 95% CI 0.067, 0.120) were independent risk factors for MALE in patients with PAD who underwent LER (Table 5).

**Table 5:** Multivariable logistic regression for MALE

	Coef.	St.Err.	t-value	p-value	[95% Conf Interval]		Sig.
Men/female	− 0.017	0.077	− 0.22	0.829	− 0.168	0.135	
Age, years	0.005	0.004	1.34	0.181	− 0.002	0.012	
Diabetes duration	− 0.016	0.037	− 0.42	0.676	− 0.089	0.058	
BMI	0.027	0.050	0.54	0.590	− 0.072	0.127	
Smoking (current)	− 0.288	0.471	− 0.61	0.542	− 1.217	0.642	
Smoking (former)	− 0.458	0.467	− 0.98	0.328	− 1.381	0.464	
Never smoked	− 0.191	0.462	− 0.41	0.680	− 1.103	0.721	
Hypertension	− 0.072	0.071	− 1.01	0.315	− 0.212	0.069	
Hypercholesterolemia	0.048	0.065	0.75	0.456	− 0.079	0.175	
CAD	0.029	0.064	0.45	0.653	− 0.098	0.156	
CVD	0.019	0.067	0.28	0.782	− 0.113	0.150	
Oral antidiabetic ag	− 0.037	0.066	− 0.55	0.580	− 0.167	0.094	
Insulin	0.151	0.074	2.05	0.042	0.006	0.297	*
ABI	0.763	0.309	2.47	0.015	0.153	1.373	*
Rutherford II-4	− 0.029	0.077	− 0.38	0.706	− 0.181	0.123	
Rutherford III-5	0.000						
WIfI010	0.026	0.100	0.26	0.795	− 0.171	0.223	
WIfI020	0.122	0.102	1.20	0.232	− 0.079	0.323	
WIfI110	0.070	0.103	0.67	0.501	− 0.134	0.274	
WIfI120	0.000						
HbA1c	0.149	0.049	3.08	0.002	0.054	0.245	**
FBG	− 0.001	0.003	− 0.30	0.765	− 0.008	0.006	
Total cholesterol	0.004	0.002	2.42	0.017	0.001	0.007	*
LDL cholesterol	0.000	0.003	− 0.07	0.944	− 0.005	0.005	
Triglycerides	0.005	0.004	1.31	0.192	− 0.003	0.012	
eGFR	0.000	0.003	0.11	0.914	− 0.006	0.006	
HMGB-1	0.093	0.013	6.95	0.000	0.067	0.120	**
Constant	− 4.110	1.918	− 2.14	0.033	− 7.894	− 0.325	*
Mean dependent var	0.463	SD dependent var	0.500				
R-squared	0.352	Number of obs	201.000				
F-test	3.807	Prob > F	0.000				
Akaike crit. (AIC)	255.362	Bayesian crit. (BIC)	341.248				

**p < 0.01

*p < 0.05

## Discussion

In this study we have evaluated the association between serum HMGB-1 levels and cardiovascular outcomes after LER in a cohort of diabetic patients with PAD and CLTI, showing an association between higher serum HMGB-1 levels and MACE and MALE during the follow-up period after revascularization.

Despite best medical therapy and a multidisciplinary approach, prevention of cardiovascular complications in T2DM patients with PAD after LER already represents an unmet need. Even though similar baseline characteristics of the population receiving LER, some patients develop no complications, while others have poor outcomes even shortly after the endovascular procedure [35].

Therefore, in addition to traditional cardiovascular risk factors, identifying predictors of adverse outcomes after LER is necessary to prevent cardiovascular events.

Inflammation is a *sine qua non* for atherosclerosis progression [43], and anti-inflammatory therapy has been demonstrated to have an adjuvant role in the secondary prevention of cardiovascular events [44–46].

The chronic inflammatory state associated with diabetes mellitus plays a key role in macrovascular and microvascular complications in diabetic patients [18, 47]. We previously showed in a cohort of 299 diabetic patients with PAD that baseline OPG, TNF-α, IL-6, and CRP levels were associated with adverse cardiovascular outcomes after LER [17]. In addition, Bleda and colleagues found associations between CRP and fibrinogen levels with mortality and cardiovascular outcomes at baseline and after LER [16].

HMGB-1 is a ubiquitous protein that acts as a pro-inflammatory cytokine in the extracellular space. It plays a crucial role in the progression of atherosclerosis and the development of cardiovascular diseases [18]. Once bound to its receptors (RAGE and TLRs), HMBG-1 activates various signaling pathways, including nuclear factor-kB (NF-kB), extracellular signal-regulated kinase (ERK), p38 mitogen-activated protein kinase (p38MAPK), c-Jun N-terminal kinase (JNK), and myeloid differentiation factor-88 (MyD88), which promote oxidative stress and enhance the secretion of various cytokines, including TNF-α, IL-6 and IL-1, growth factors and adhesion molecules, contributing to endothelial dysfunction and vascular injury [18]. HMGB-1 has been shown also to promote vascular endothelial growth factor (VEGF) dependent angiogenesis in animal models of limb ischemia, suggesting a role of HMGB-1 in tissue repair [48, 49].

Higher HMGB-1 levels are associated with poor outcomes after myocardial infarction (MI) [50–52]. Specifically, Kohno and colleagues showed that patients with ST-elevation myocardial infarction (STEMI) had higher serum HMGB-1 levels at baseline and 12 h after the event than patients with chronic stable angina, and this was associated with adverse cardiovascular outcomes, in particular pump failure, cardiac rupture and cardiac death [50]. SØrensen and colleagues showed an association between higher HMGB-1 levels and increased mortality after STEMI [51]. Hashimoto and colleagues found similar results in a cohort of patients with unstable angina and non-ST-segment elevation myocardial infarction (NSTEMI), suggesting an association between higher HMGB-1 levels and risk of cardiovascular death [52]. We previously showed that elevated serum HMGB-1 levels were associated with the presence and the severity of PAD in diabetic patients, compared with diabetic patients without PAD [33]; however, an association between HMGB-1 and poor cardiovascular and limb outcomes after LER has not been demonstrated.

In the present study, we found that serum HMGB-1 levels in diabetic patients with CLTI were associated with MACE after LER. In particular, patients with higher levels of HMGB-1 had a higher risk of myocardial infarction, stroke and death from cardiovascular diseases. Smoking status and higher triglycerides levels were also associated with MACE, in line with previous findings [53]. In addition, non-smokers and former smokers were less likely to develop a MACE. Interestingly, there were no differences in the incidence of MACE among patients with and without history of CAD.

The relationship between HMGB-1 levels and MACE persisted even after adjusting for traditional cardiovascular risk factors such as age, BMI, smoking habits, hypertension, blood lipids, glycemic control and renal function, confirming HMGB-1 as an independent risk factor. Interestingly, ABI was also associated with MACE in the multivariable analysis, supporting the results of previous work by Mendes-Pinto and colleagues [54]. Surprisingly, traditional cardiovascular risk factors did not appear to play a role in cardiovascular complications after LER. A history of CAD was not associated with MACE in multivariable analysis too. The results may be due to the small sample size of our cohort, perhaps the intrinsic advanced disease bias of our cohort, and the short follow-up time. The finding, however, may also be representative of a distinctive pro-inflammatory state after peripheral vascular revascularization in which HMGB-1 may have a key role in clinical outcome prediction. Specifically, we elaborated a ROC curve that included only HMGB-1, confirming the role of HMGB-1 in predicting MACE after LER, and a second ROC curve that included serum HMGB-1 levels and traditional cardiovascular risk factors. Comparing these ROC curves, we found that the model with HMGB-1 and traditional cardiovascular risk factors improved the prediction of MACE.

Interestingly, it has been demonstrated that HMGB-1 levels were associated with a number of diseased coronary vessels in patients with acute coronary disease, confirming the role of HMGB-1 in determining MACE [55]. In our study, however, we did not evaluate the extension of diseased coronary vessels given the design of the research that aimed at evaluating HMGB-1 levels as predictor of MACE, regardless of the extension of CAD.

An additional novelty of our study is that we found a cut-off value for HMGB-1 that could predict MACE free survival after endovascular revascularization. HMGB-1 levels above 6.905 ng/mL predict a high risk of MACE with a good sensitivity (69.14%) and specificity (78.33%).

Another finding of our study was that higher HMGB-1 levels were even associated with MALE after LER. Compared with patients without MALE, patients with MALE were active smokers, were on insulin and had higher levels of total cholesterol and triglycerides. Conversely, non-smokers were less likely to develop a MALE.

The association between triglycerides and other cardiovascular risk factors with MALE was attenuated in our multivariable logistic regression after adjusting for HMGB-1, which was confirmed to be an independent risk factor for MALE, along with the use of insulin, ABI, glycated hemoglobin and total cholesterol levels, consistent with earlier evidence [56, 57]. Finally, the role of HMGB-1 as a predictor of MALE was demonstrated by a ROC curve.

Various explanations would support our results. In fact, an important pro-atherogenic role of HMGB-1 has been demonstrated in the literature [58–60]. HMGB-1 acts by regulating various inflammatory factors and immune cells; it also promotes the recruitment of vascular smooth muscle cells (VSMCs) and foam cells in atherosclerotic lesions [58]. VSMCs themselves enhance HMGB-1 secretion in an autocrine loop, which may lead to neointimal hyperplasia responsible of restenosis after angioplasty [58]. Furthermore, Wang and colleagues demonstrated that LPS/ATP activation of NLRP3 inflammasome stimulates HMGB-1 release leading to cholesterol accumulation in VSMCs and foam cells formation during atherosclerosis, through the downregulation of LXRa and ABCA1 expression [60]. HMGB-1 is even responsible for atherosclerotic plaque vulnerability, possibly through VSMCs secretion of matrix metalloproteinase (MMP), enzymes responsible for matrix degradation [32, 61].

Interestingly, high expression of HMGB-1 was found in platelet-rich coronary thrombus from MI patients [62], suggesting that activated platelet-derived HMGB-1 may represent a link between atherothrombosis and inflammation [63]. In fact, activated platelets have been found to be the major source of HMGB-1 in thrombi, and activated platelet-derived HMGB-1 has been shown to play a key role in small vessels thrombosis, by promoting platelet aggregation, platelet granule secretion, and immune cells recruitment [63]. Moreover, a study by Lv and colleagues showed that HMGB-1 is able to induce tissue factor expression in endothelial cells and macrophages through TLR4, TLR2, RAGE receptors and through transcriptional factors, such as NF-kB and Egr-1, favoring atherothrombosis [64]. A similar result was found by Sugimoto and colleagues that demonstrated an enhanced tissue factor expression by HMGB-1 in human aortic endothelial cells [65].

The evidence discussed supports our findings and indeed HMGB-1 may be an essential mediator of acute cardiovascular outcome after LER and in acute limb adverse events after revascularization.

This study, however, has several limitations. First, this is a single-center study based only on the Italian population and the cohort analyzed was relatively small. Therefore, the results cannot be applied to other ethnic groups and must be validated in larger patient groups. The small sample size may be the reason why the multivariable analysis failed to capture the effects of traditional risk factors on MACE and MALE, along with the short follow-up time of observation.

Furthermore, we did not assess levels of other cytokines, so we could not conclude whether the association between HMGB-1 and poor adverse events after LER remained significant after adjusting for other inflammatory parameters.

Another limitation of the study is that we did not evaluate changes in HMGB-1 levels during follow-up after LER and their impact on cardiovascular adverse events. Our goal, however, was to identify a biomarker that at baseline could predict poor outcome after revascularization.

## Conclusion

To the best of our knowledge, this study demonstrates a novel association between baseline serum HMGB-1 levels with MACE and MALE in diabetic PAD patients with CLTI after LER. This association furthers the understanding of the key role endothelial mediated inflammation plays in post-revascularization cardiovascular complications and may highlight a distinctive mechanistic pathway that can facilitate the development of novel personalized strategies to prevent cardiovascular events.

## Data Availability

The datasets generated during the current study are available from the corresponding author on reasonable request.

## Abbreviations

DM: Diabetes mellitus
PAD: Peripheral arterial disease
MACE: Major cardiovascular events
MALE: Major adverse limb events
LER: Lower-extremity endovascular revascularization
CLTI: Chronic limb-threatening ischemia
CRP: C-reactive protein
OPG: Osteoprotegerin
TNF: Tumor necrosis factor
IL: Interleukin
HMGB-1: High mobility group box-1
NK: Natural killer
LPS: Lipopolysaccharide
IFN: Interferon
RAGE: Receptor for advanced glycation end products
TLR: Toll-like receptor
CAD: Coronary artery disease
ABI: Ankle/brachial index
US: Ultrasound Color Doppler
WIfI: Wound, Ischemia, and foot Infection
BMI: Body mass index
CVD: Cerebrovascular disease
eGRF: Estimated glomerular filtration rate
DAPT: Double antiplatelet therapy
MDRD: Modification of diet in renal disease
ROC: Receiver operating characteristics
FBG: Fasting blood glucose
NF-kB: Nuclear factor-kB
ERK: Extracellular signal-regulated kinase
p38MAPK: P38 mitogen-activated protein kinase
JNK: C-Jun N-terminal kinase
MyD88: Myeloid differentiation factor-88
VEGF: Vascular endothelial growth factor
MI: Myocardial infarction
STEMI: ST-elevation myocardial infarction
NSTEMI: Non-ST-segment elevation myocardial infarction
VSMCs: Vascular smooth muscle cells
MMP: Matrix metalloproteinase
